# Age-related mating rates among ecologically distinct lineages of bedbugs, *Cimex lectularius*

**DOI:** 10.1186/s12983-023-00505-z

**Published:** 2023-07-28

**Authors:** Tomáš Bartonička, Jana Křemenová, Ondřej Balvín, Zdeněk Šimek, Oliver Otti

**Affiliations:** 1 Department of Botany and Zoology, Faculty of Science, Masaryk University, Kotlářská 2, 611 37 Brno, Czech Republic; 2 Department of Ecology, Faculty of Environmental Science, Czech University of Life Sciences Prague, Kamýcka 129, 165 21 Prague 6, Czech Republic; 3 Research Centre for Toxic Compounds in the Environment, Masaryk University, 62500 Brno, Czech Republic; 4 Animal Population Ecology, Animal Ecology I, University of Bayreuth, Universitätsstrasse 30, 95440 Bayreuth, Germany

**Keywords:** Bedbugs, Mating scars, Reproduction, Sexual conflict, Age, Pteridines

## Abstract

Understanding how many mates an animal has in its lifetime is a critical factor in sexual selection. At the same time, differences in an organism's ecology, such as the quantity and quality of food, could be reflected in different mating rates. Mating rate had a significant effect on female net fitness (i.e., lifetime offspring production), however, laboratory measurements cannot well mirror the situation in wild. The common bedbug (*Cimex lectularius*) is a well-established model for studying traumatic insemination and sexual conflict. The species comprises two host lineages that feed on bats (BL) or humans (HL). HL can constantly feed on human hosts throughout the year, while BLs feed only during summer months when their bat hosts occupy the roosts. Because mating in female bedbugs is closely linked to foraging, this system provides a valuable model to study mating variation in the field. We established a new method for estimating age-dependent mating rates of females in the wild by relating the fluorescent pigment accumulation in the eyes of females to the number of mating scars that manifest as melanized spots caused by the injection of sperm through the wall of the female abdomen by the male into the spermalege. In addition, using laboratory bedbugs we found that three and a half observed matings on average lead to one observed melanized mating scar. Although young BL and HL females (with low pteridine concentrations) did not differ in the number of matings, the mating rate increased with age only in HL but not in BL females. We sampled on average older BL than HL females. The lack of access to food (bat blood) during winter could explain the lack of increase in the number of scars with age in BL. In species where mating leaves visible marks, using fluorescent pigments to determine female age (applicable to most arthropods) could be an important tool to study sexual selection and mating rate in the wild. The method can help formulate sustainable and biologically lucid approaches for their control.

**Supplementary Information:**

The online version contains supplementary material available at 10.1186/s12983-023-00505-z.

## Introduction

Populations of the same species may differ in many aspects, such as density, presence of predators and pathogens, or other ecological variables [14, 49]. Intraspecific variation in reproductive behaviour between populations has been widely documented (e.g., [35, 43, 45]). Differences among populations could influence mating systems such as intrasexual competition, female choice or resistance, optimal mating rates [22], or even lead to reproductive isolation [32].

The willingness of females to mate depends on several factors, particularly female/male size [44], female age [12], male quality [33], but also food availability and composition [17, 51]. Measuring mating rates in the laboratory can often overestimate the natural situation, as laboratory females may not be able to avoid mating in artificial and often small cages [1] with limited dispersal and hiding possibilities. If the female cannot escape the persistent male, she usually has no choice and is mated repeatedly. Compared to the limited escape opportunities in the laboratory, a rugged natural environment increases the likelihood that females will avoid multiple mating reducing their longevity and reproductive success [47]. Direct observations of successive copulations in the field are time consuming or technically impossible [15, 24, 48]. Assessing the presence/absence of spermatophores among insect females (Lepidoptera, Coleoptera, Heteroptera) sampled in a population is probably the most common method [7, 26]. In addition, it only provides information on the number of males involved in fertilisation (inferred from mother–offspring analyses of a polymorphic microsatellite locus) and not directly on the actual mating rate [13, 15, 21]. Other methods for estimating mating frequency in the field exist but have many limitations (reviewed in [37].

Here, we consider the mating frequency of males and females in two lineages of the common bedbug (*Cimex lectularius* Linnaeus, 1758), one feeding on blood of bats (BL) and other on human blood (HL) [3]. Females must feed regularly to produce eggs, and during feeding their body volume increases substantially. Reinhardt et al. [40] have shown that fully fed females attract more mating attempts, being also less resistant to mating. If females are indeed unable to resist mating in the wild, we expect strong differences in mating rates between HL and BL. While humans provide a stable food source through the whole year, bats leave their summer roosts for the whole winter, often for more than 6 months.

This study aimed to test whether we can measure the mating rate in the wild by calibrating the number of mating events with mating scars and using age estimation by pteridine concentration in a field population. Therefore, we have developed laboratory tests to determine the relationship between the number of matings and the number of scars. Furthermore, we tested how well our methods work, and therefore we examined two lines of bugs that differ in their ecology and thus potentially in mating rate and/or age structure. We predicted that host availability (HL versus BL) and host abundance (in BL) would be correlated with the mating rate.

## Material and methods

### Bedbug laboratory culture

Human-associated (HL) *Cimex lectularius* was sourced from a large laboratory population at the University of Bayreuth Germany, originally collected in London (UK) in 2008 (reared for approximately 50 generations). All bedbugs were maintained in an incubator at 27 ± 1 °C with a 12h/12h L:D light regime at 70% relative humidity. Feeding and grooming protocols follow Reinhardt et al. [39] and Kaldun and Otti [18].

### Laboratory calibration of mating scars

Wounding by traumatic insemination (TI) activates phenoloxidase and induces the formation of melanin in the injured tissues, leaving a mating scar [19, 20, 30] that becomes fully visible 12 h after mating (Additional file 1: Figs. S1, S2).

We produced 160 adult virgin females of four different age groups, i.e., 10, 23, 34, and 62 days old (n = 40 each). Following our previous study [23], these age groups were selected to control for a possible age effect on melanisation. Previous studies have suggested that light exposure influences pteridine accumulation [27, 29]. Therefore, half of the adult females were kept in constant darkness and the other half in a 12h:12h light:dark cycle (20:20 females per age group). We then randomly assigned five virgin females from each age group and lighting treatment to each of the four different mating treatments, i.e., five, ten, fifteen, and twenty matings, respectively. For the matings, females were placed individually in plastic Petri dishes (diameter 55 mm) provided with filter paper, then a male was added, and the entire copulatory sequence was observed. The sperm is received in the spermalege, a paragenital organ evolved to decrease costs of wounding inflicted in mating [39]. For every male, we carefully checked if it made the intromission movement of the paramere and if it kept still on the female afterwards, i.e., the behaviour indicating sperm transfer. In addition, we visually inspected if sperm was transferred after each mating. Sperm transfer can be easily observed under the cuticle of the female. Males were randomly selected from a pool of 200 males, that were also virgin by the beginning of the experiment. During the experiment, males were reused for more than one mating because we were only interested in the scars inflicted by TI. Once a male completed a mating, it was placed in a new holding container to replenish seminal fluid and was not reused until all remaining males were depleted. Matings were distributed over five days to reduce any possible influence of time since the last feeding, i.e., females in the four mating groups were mated once, twice, three times, and four times per day, respectively. After the last mating, females were kept individually in a Drosophila vial with a piece of filter paper for two days to allow for melanisation, i.e., the mating scar, to become visible [34, 42, 50]. Then, females were dissected under a microscope and the number of mating scars was counted on the inner part of the spermalege.

### Statistical analysis of the calibration of mating scars

All statistical analyses were performed with R 4.0.3 [36] using the package car [11]. Nine females died during the experiment (6 in the fifteen matings group and 3 in the twenty matings group) and were excluded from the analysis. We fitted two generalised linear models (GLM) to analyse the age and lighting effect on i) the number of mating scars and ii) the number of scars per mating. In these models, we fitted the number of matings as a continuous variable to characterise the relationship between the number of scars and matings. We extracted F-statistics from both models using the anova() function and checked for normality and homogeneity in both analyses by visually inspecting the residuals compared to the fitted plots and using the qqnorm() function.

### Bedbug sampling in the field

We conducted one-time collections in 13 bat colonies in the Czech Republic (mid-June 2018), resulting in 292 randomly collected BL females. BL were sampled between 30 and 40 days after the arrival of most females to the roost of each bat colony. We also collected 216 HL females by one-time visits of 13 human infestations across Europe (collected between 2006 and 2014) (Table 1). To assess food availability and differences between nursery colonies, numbers of bats were recorded during bedbug sampling. The number of bats has been shown to positively correlate with the number of bedbugs [2]. But it is unclear whether more bedbugs can lead to a higher mating rate, i.e. a higher number of scars. The number of bats in colonies was estimated by experienced members of the Czech Bat Conservation Society and have been refined from photographs.

**Table 1:** Number of collected females, used for pteridine extraction (extracted), with countable scars used in mating rate analysis (material)

Site	Collected	Extracted	Countable scars	Material	Iso concentration			
					Mean	SD	D10	D90
*BL*								
Bila Lhota	15	15	15	15	8.0	8.0	1.1	21.8
Bucovice	20	19	20	19	35.4	14.8	8.9	48.2
Hanusovice	22	22	18	18	16.8	6.6	8.5	24.1
Loukov u Semil	30	30	23	23	24.9	16.5	9.2	51.1
Moravicany	30	21	26	20	20.0	13.2	4.8	38.0
Mostkov	28	23	27	22	21.3	16.1	4.0	44.8
Otaslavice	25	25	23	23	62.5	19.4	4.7	83.3
Raskov	21	21	20	20	18.7	12.4	5.5	37.6
Snedovice	15	14	14	13	11.9	9.0	4.2	24.4
Ustek	25	25	20	20	14.1	6.0	5.8	21.5
Veselicko	21	21	20	20	14.8	63.3	5.8	225.4
Viszlo, Hungary	15	15	15	15	23.1	10.4	8.3	37.2
Vysoke Veseli	25	24	25	24	109.4	56.8	4.1	207.5
All	292	275	266	252	42.0	49.0	5.3	99.2
*HL*								
Bohumin—Cs. armady	23	23	21	21	4.5	5.2	1.4	9.8
Bohumin—Okruzni	11	11	11	11	28.9	29.9	4.7	54.3
Havirov—Senov	25	25	22	22	24.8	10.4	13.9	41.6
Havirov—Sumbark	11	11	11	11	17.9	8.1	11.1	28.3
Krakow, Poland	20	20	16	16	3.7	0.7	2.9	4.9
Melk, Austria	22	22	21	21	46.6	32.6	5.7	79.1
Ostrava—Valcovny	9	9	9	9	1.9	1.1	1.0	4.3
Ostrava—Zabreh	18	18	18	18	9.5	4.7	3.4	14.6
Ostrava—Fifejdy	19	18	18	17	27.0	18.7	5.5	55.5
Rokycany	25	25	22	22	26.8	17.0	12.6	48.3
Schaffhausen, Switzerland	11	11	11	11	19.1	13.2	4.9	27.5
Straz pod Ralskem	11	11	11	11	152.3	94.4	12.8	249.0
Venice, Italy	11	11	10	10	18.6	17.4	4.0	43.6
All	216	215	201	200	27.5	42.1	2.2	64.0

Site	Scars										Colony size
	Mean	SD	Min	Max	D10		D90		Q1		
					Mean	SD	Mean	SD	Mean	n	
*BL*											
Bila Lhota	1.5	2.3	0.0	8.0	1.0	1.4	3.0	2.8	1.3	10	370
Bucovice	1.6	2.2	0.0	9.0	6.0	4.2	2.5	2.1	3.2	2	280
Hanusovice	3.4	2.6	0.0	9.0	1.5	0.7	2.0	1.4	3.2	3	1037
Loukov u Semil	0.6	1.4	0.0	5.0	0.3	0.6	2.3	2.5	0.8	6	880
Moravicany	3.7	3.3	0.0	10.0	3.5	3.5	4.0	0.0	6.2	6	1029
Mostkov	2.0	1.6	0.0	5.0	1.0	1.0	3.0	2.0	2.5	10	92
Otaslavice	2.6	2.7	0.0	10.0	2.7	2.5	4.0	1.0	2.8	1	295
Raskov	5.5	2.4	2.0	9.0	6.0	4.2	5.5	5.0	4.6	8	1916
Snedovice	3.0	1.5	0.0	5.0	2.0	2.8	2.5	0.7	2.8	8	326
Ustek	3.1	2.5	0.0	7.0	0.5	0.7	5.0	2.8	1.4	4	972
Veselicko	2.3	2.0	0.0	7.0	4.5	3.5	0.5	0.7	3.4	1	669
Viszlo, Hungary	3.9	2.4	1.0	9.0	3.5	3.5	3.0	1.4	2.3	3	370
Vysoke Veseli	1.1	1.3	0.0	4.0	1.0	1.7	0.3	0.6	1.7	1	600
All	2.6	2.5	0.0	10.0	2.5	3.0	1.5	1.6	2.9	63	
*HL*											
Bohumin—Cs. armady	3.7	3.6	0.0	17.0	1.5	2.2	4.3	1.2	–	–	–
Bohumin—Okruzni	4.1	2.6	0.0	7.0	3.0	2.8	5.5	0.7	–	–	–
Havirov—Senov	5.2	3.3	0.0	11.0	4.0	3.5	4.3	2.5	–	–	–
Havirov—Sumbark	5.5	2.8	2.0	10.0	3.5	2.1	3.5	0.7	–	–	–
Krakow, Poland	2.1	1.7	1.0	6.0	3.5	3.5	1.0	0.0	–	–	–
Melk, Austria	1.0	1.4	0.0	5.0	1.0	0.0	2.0	2.7	–	–	–
Ostrava—Valcovny	1.1	1.1	0.0	3.0	2.5	0.7	0.0	0.0	–	–	–
Ostrava—Zabreh	1.7	1.4	0.0	6.0	0.5	0.7	3.5	3.5	–	–	–
Ostrava—Fifejdy	2.5	1.9	0.0	6.0	0.0	0.0	3.5	0.7	–	–	–
Rokycany	6.2	3.6	0.0	15.0	7.0	3.5	7.3	3.8	–	–	–
Schaffhausen, Switzerland	7.4	3.0	2.0	12.0	6.5	6.4	7.5	0.7	–	–	–
Straz pod Ralskem	4.4	3.0	1.0	9.0	4.5	2.1	6.5	2.1	–	–	–
Venice, Italy	3.6	1.2	2.0	6.0	2.0	0.0	4.0	0.0	–	–	–
All	3.7	3.2	0.0	17.0	2.4	3.8	3.4	3.3	–	–	–

Mean, standard deviation (SD), 10% and 90% quantile (D10, D90) of isoxanthopterin concentration (*Iso* concentration). Mean, SD, minimum, maximum of number of mating scars (Scars), mean and SD for the youngest (D10) and oldest (D90) females, mean and number of young BLs with Iso concentration lower than 25% quantile (Q1). All variables are for each sampled locality and for whole bat- and human-associated lineages (All BL and All HL). Sizes of nursery colonies are added for BL localities (Colony size). All sites, unless specified, are in the Czech Republic

### Age analysis by pteridine concentration

Pteridine extraction was performed according to the protocol published in Křemenová et al. [23]. Briefly, bedbugs were decapitated, and the head capsules were homogenized separately using a microtissue grinder with 200 μL of buffer and the suspension was transferred to a vial. The vials containing the suspension were left in an ultrasonic bath for approximately two hours and then centrifuged at 6000 rpm for 5 min. 0.5 ml was transferred to a sealed dark glass vial and stored at − 20 °C until liquid chromatography-tandem mass spectrometry (LC–MS/MS) was performed. Following Křemenová et al. [23], we chose isoxanthopterin (CAS 529-69-1) as the standard for LC–MS/MS analysis, which was purchased from Sigma Aldrich Corporation (St Louis, MO, USA).

Liquid chromatograph Agilent 1290 Infinity II Series (Agilent Technologies, Santa Clara, CA) was used to separate isoxanthopterin from other pteridines. For separation, we used a Luna NH2 chromatographic column (100 Å, 150 × 2.0 mm 3 μm, Phenomenex, USA) at a column temperature of 30 °C. The injected sample volume was 5 µl (for more details see [23].

We were able to extract pteridines from 490 females to estimate the age distribution. The age status of females is represented by isoxanthopterin (Iso) concentration, with Iso concentration increasing with female age [23]. Only 467 females had visible and countable mating scars and were therefore used to estimate mating rates.

### Statistical analysis of mating rate in the wild

All statistical analyses were performed with R 4.0.3 using the lme4 (Bates et al., 2015) and lmerTest [25] packages. For analysis of the number of mating scars, we fitted a generalized linear mixed effects model (GLME) with Poisson distribution with lineage (BL, HL) and age (log Iso concentrations) as fixed factors and population as a random effect. We checked normality and homogeneity in both analyses by visually inspecting the residual versus fitted plots and the qqnorm() function. We used the Wilcoxon test to compare the mean values of Iso concentration and number of scars.

## Results

### Laboratory calibration of mating scars

**Fig. 1:**
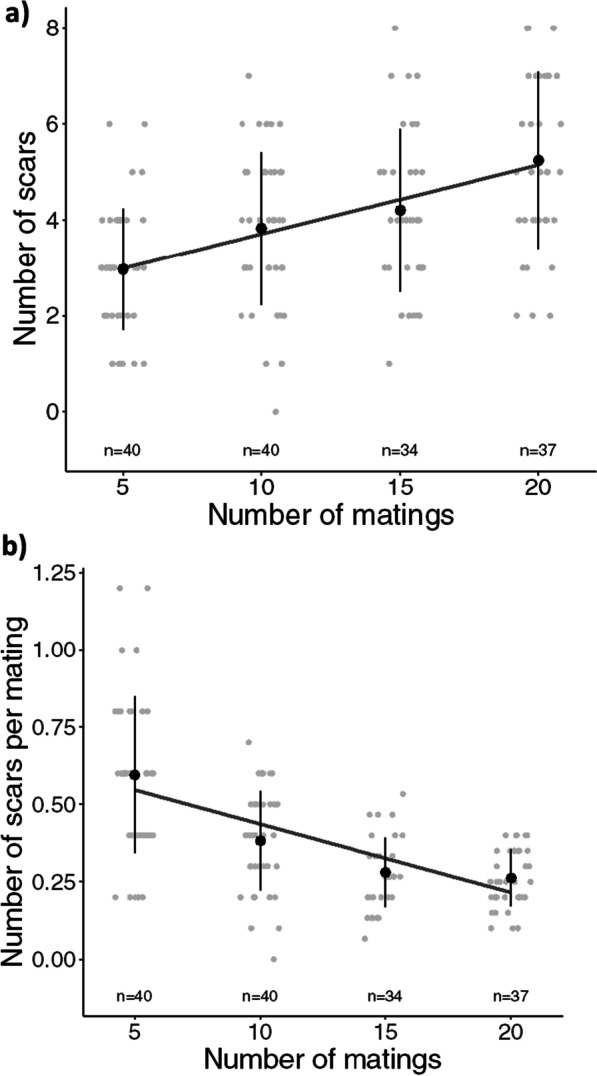
Number of mating scars (**a**) and number of scars per mating (**b**) measured for four mating groups of females mated with 5, 10, 15 or 20 males. Error bars represent one standard deviation, black points correspond to means, grey points are individual data points, and the dark grey line indicates the linear relationship between the number of scars and the number of matings

Neither female age (ANOVA: F_1,147_ = 1.966, p = 0.163) nor light conditions (ANOVA: F_1,147_ = 0.064, p = 0.801) affected the number of mating scars. The number of mating scars increased significantly with the number of female matings (ANOVA: F_1,147_ = 37.869, p < 0.0001) (Fig. 1a). When we looked at the number of scars per mating, we again found no effect of female age (ANOVA: F_1,147_ = 1.037, p = 0.310) or light conditions (ANOVA: F_1,147_ = 0.054, p = 0.816). However, the number scars per mating significantly decreased with the number of matings (ANOVA: F_1,147_ = 75.602, p < 0.0001) (Fig. 1b).

Furthermore, the minimum number of matings producing at least one scar (total number of matings/total scars) averaged 3.5 ± 2.2 (mean ± SD, n = 150 females) matings across all treatment groups. At low mating numbers (> 5), the ratio of matings to scars is almost 1:1, while at 15 and 20 matings the ratio of matings to scars is similar and approaches 3:1 (Fig. 1b).

### The mating rate in the wild

**Fig. 2:**
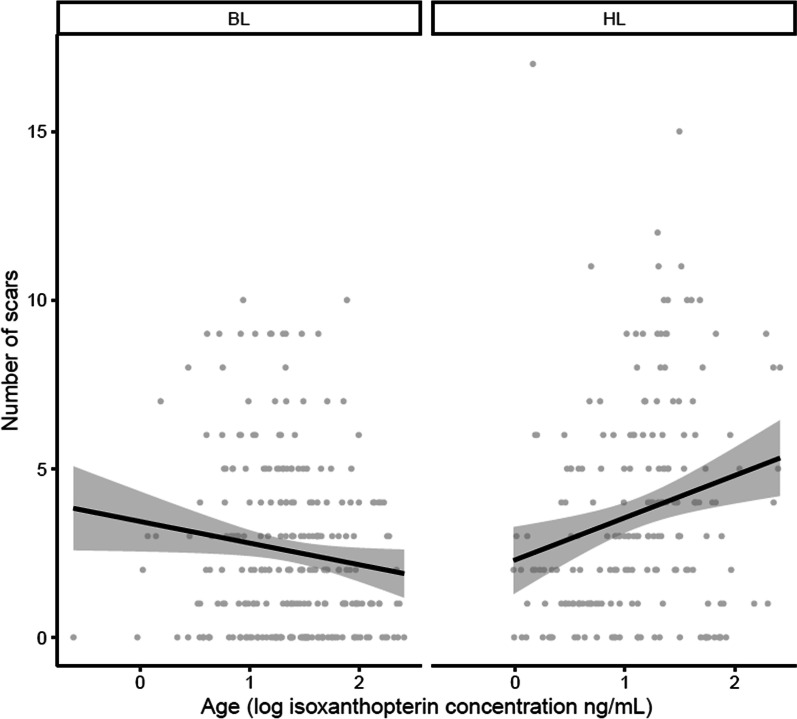
Relationship between the number of scars and isoxantopterin concentration in bat-associated (BL) and human-associated (HL) populations. Grey points show individual females and the black line represent the linear regression between number of scars and isoxanthopterin concentration with the 95% confidence interval shaded in grey

In wild populations, we found a relatively low number of scars (max = 10 in BLs, max = 17 in HLs), but usually less than five scars. The number of scars in wild-collected females was significantly affected by the interaction between female age (represented by the logarithm of Iso concentration) and origin (GLME with Poisson distribution: $\chi_{{{1},{452}}}^{2}$ = 9.99, p = 0.002). In other words, the number of mating scars decreases with age in BLs, while an opposite trend is observed in HLs, where the number of scars increases with age (Fig. 2).

Moreover, we can observe that although the BL females in our samples are older (the mean value of Iso concentration is higher; Wilcoxon test: Z_1,452_ = 4.81, p < 0.0001), they have a lower number of scars than HLs (Wilcoxon test: Z_1,452_ = -3.83, p < 0.0001). The low 10% quantile of BL females had the same number of scars as the low 10% quantile (D10) in HL females (HL mean 2.4 ± 3.8, n = 21 vs BL mean 2.5 ± 3, n = 26), but the 90% quantile (D90) was in HLs higher than in BLs (HL mean 3.4 ± 3.3, BL mean 1.6 ± 1.5, Table 1).

The number of matings in young BLs (lower 25% quantile of Iso concentration, Q1, n = 63) was significantly dependent on bat colony size, but not on female age (GLM with Poisson distribution: log Iso concentration: χ_1.62_^2^ = 0.86, p = 0.354; Colony size: χ_1.62_^2^ = 13.59, p < 0.001). Low Iso values (< Q1) based on laboratory measurements [23] correspond to age cohorts of bedbugs (< 107) before the bats leave their roosts.

## Discussion

We have shown experimentally that the number of melanized scars correlates with the number of matings, although not in a 1:1 ratio. Therefore, we expected that every intromission leads to a puncture of the cuticle and leaves a mating scar. However, at higher mating numbers (> 10) the probability of detecting a new scar decreased. One possible explanation could be that the more scars already present, the more likely a male pierces just next to or into older scars. Future studies could examine both the maximum number of mating scars observed and the actual intromission into mating scars by fixing mating pairs with liquid nitrogen. Regardless of the exact relationship, however, we show that more matings result in more mating scars, even at higher mating numbers. In only few cases (only in two females), we found fewer matings than scars. One explanation for these observations could be that males pierce several times during mating when females shake off males. However, we only observed this at the lowest number of matings (~ 5) in the laboratory (Fig. 1a).

### Mating rate differences between lineages

In wild populations, we found a relatively low number of scars, usually less than five scars. Our laboratory data suggest that at these low numbers, matings and scars are well correlated (Fig. 1a) and not underestimated. Considering that HL females in human infestations appear to feed approximately every 5–10 days [40], and assume that the Iso concentration values (D10, Table 1) correspond to a female age < 25 days [23], we would expect ~ 5 mating scars, respectively > 15 matings. However, the fact that scar numbers are less than half in wild HLs (2.4) indicates the ability of females to avoid mating even under high food availability.

HL and BL bedbugs with low ISo concentrations (< D10) probably only fed once or only a few times and had a similar number of scars (and thus mating rate). In addition, we found a different number of scars in old females (> D90), while BL had significantly fewer scars. This difference may be due to differences in food availability. We observed that HL females with constant access to food are unable to resist mating, and their mating scars accumulate over time. In contrast, BL females that cannot mate during a large part of the year when bats are out of the roost and unavailable as hosts had little or no accumulation of mating scars. Moreover, based on rearing experience (Sasínková et al. submitted), they feed less frequently than HL females (usually every 14 days) and thus the number of scars is more consistent with our findings from the laboratory. Multiple mating is costly and affects female fecundity and longevity [5], 39]. This cost might be reflected in the higher proportion of old females appeared in BL than in HL. More old females of BL indicate higher survival during sporadic food intake caused by the absence of a host in the shelter, at the same time a higher proportion of old females allows the survival of the period without a host and the establishment of a new population after its arrival.

Males are also limited in their ability to mate multiple times. Previous studies [18, 41] have shown that HL males need two weeks (two feedings) to replenish their seminal fluid stores and therefore cannot mate as often as HL female feed. It is not known whether there are differences in the recovery rate of ejaculate stores (sperm and seminal fluid) between BL and HL males. However, even in small laboratory populations, males would not mate to the point of ejaculate depletion [41] and one should not always expect the highest possible male mating rate. Unfortunately, no data on feeding frequency for BL males exists.

Further study should disentangle if the differences in scar numbers are related exclusively to the number of feedings or to the ability to avoid mating actively. The feeding frequency of marked bedbug females could be checked at regular intervals. Such observations of feeding frequency of BL would be possible in bat boxes (which are demountable, [4]) during the night when bats are foraging and therefore not roosting in the boxes.

### Mating rate differences within BL lineage

We have shown that the number of scars increases with the number of bats in the nursery colonies when bats are present in the roosts. For this analysis, we selected females with Iso concentration less than Q1, i.e. < 107 days. This age corresponds roughly to the time bats are present in the nursery colony roost between May and August, where they give birth and care for the young until they become fledged. We hypothesize that if more bats are at the roost, the BL females have more opportunity to feed leading to a higher mating frequency. Consequently, they should have more mating scars. As bats frequently change sites in the large attics with respect to day temperature changes, i.e. overheating [52, 53], especially the less numerous colonies become a less available food source for bed bugs than a human who does not move from his bed during the night. If, on the other hand, the colony is very large and has only limited attic space, the bedbugs have good access to the host and can feed regularly, which is related to our finding of a positive correlation between the number of bats in the colony and the number of mating scars found in female bedbugs. Moreover, in the presence of many males, males exhibit shorter latencies to mount a female than when fewer males are present [9].

On the other hand, low dietary availability, i.e., the inability to increase body size after feeding also reduced male mating attempts in bedbugs [40]. Reduced mating rates were also found in populations of other insects when access to meal was limited [28, 31]. Therefore, we suggest that the number of mating scars can be used as a proxy variable to determine mating rates in insects with variable access to food.

## Conclusion

In summary, our study introduces a new approach to evaluating age-corrected mating rates in the field. It demonstrates its potential to compare changes in mating rates over the life course of females. However, some important information as feeding frequency in the wild is still missing.

Although TI has come to the fore in studies of Heteroptera, mating scars are common throughout the animal kingdom [38]. In established insect models such as Coleoptera [8, 10, 16] or Diptera [6, 19, 46], our method makes mating rate estimation applicable in the wild.

## Supplementary Information

**Additional file 1: Supplementary methods:** Melanisation of mating scars over time.** Supplementary figures: Figure S1.** Mating scars on the female ectospermalege.** Figure S2.** Melanization of mating scars over time.

## Data Availability

All data are part of the paper or Additional file 1: Appendix. Sampling locations, numbers of scars, bats, *Iso* concentrations data: Figshare https://doi.org/10.6084/m9.figshare.18592895.v1.
