# Supplementary material for: Age-related mating rates among ecologically distinct lineages of bedbugs, Cimex lectularius
Source: Front Zool. 2023 Jul 28;20:25. doi: 10.1186/s12983-023-00505-z (PMC10375771; doi:10.1186/s12983-023-00505-z)
Supplement: Supplementary file 1 — Additional file 1: Supplementary methods: Melanisation of mating scars over time. Supplementary figures: Figure S1. Mating scars on the female ectospermalege. Figure S2. Melanization of mating scars over time. [file 12983_2023_505_MOESM1_ESM.doc]

**APPENDIX**

**Age-related mating rates among ecologically distinct lineages of bedbugs, *Cimex lectularius***

Tomáš Bartonička^1^*, Jana Křemenová^1^, Ondřej Balvín^2^, Klaus Reinhardt^3^, Zdeněk Šimek^4^, Oliver Otti^5^

^1^Department of Botany and Zoology, Faculty of Science, Masaryk University, Kotlářská 2, 611 37 Brno, Czech Republic, ^2^Department of Ecology, Faculty of Environmental Science, Czech University of Life Sciences Prague, Kamýcka 129, 165 21, Prague 6, Czech Republic, ^3^Applied Zoology, Department of Biology, Technische Universität Dresden, 01062 Dresden, Germany, ^4^Masaryk University, Research Centre for Toxic Compounds in the Environment, Brno, 62500, Czech Republic. ^5^Animal Population Ecology, Animal Ecology I, University of Bayreuth, Universitätsstrasse 30, 95440 Bayreuth, Germany

Supplementary methods

Melanisation of mating scars over time

Supplementary figures

Figure S1 – Mating scars on the female ectospermalege

Figure S2 – Melanization of mating scars over time

# Supplementary methods

## Melanisation of mating scars over time

We mated 120 females from the same population to establish the time point when a mating scar becomes visible. Matings were interrupted after 60s to standardize for copulation duration across males. After each of six different time points, i.e. 30 minutes, 1 hour, 12 hours, 24 hours, 48 hours and five days, we transferred 20 females to EtOH (99%) until further processing. Once all females had been transferred to a 1.5ml Eppendorf tube containing absolute EtOH they were dissected under a stereo microscope and the inner part of the spermalege was photographed with a digital imaging system (Leica DFC290 and Leica Application Suite software, version 2.7.1 R1 [Build: 1384], Leica Microsystems). The person counting mating scars was blind with respect to the time point of the female transfer to a 1.5ml Eppendorf tube containing absolute EtOH.

# Supplementary figures


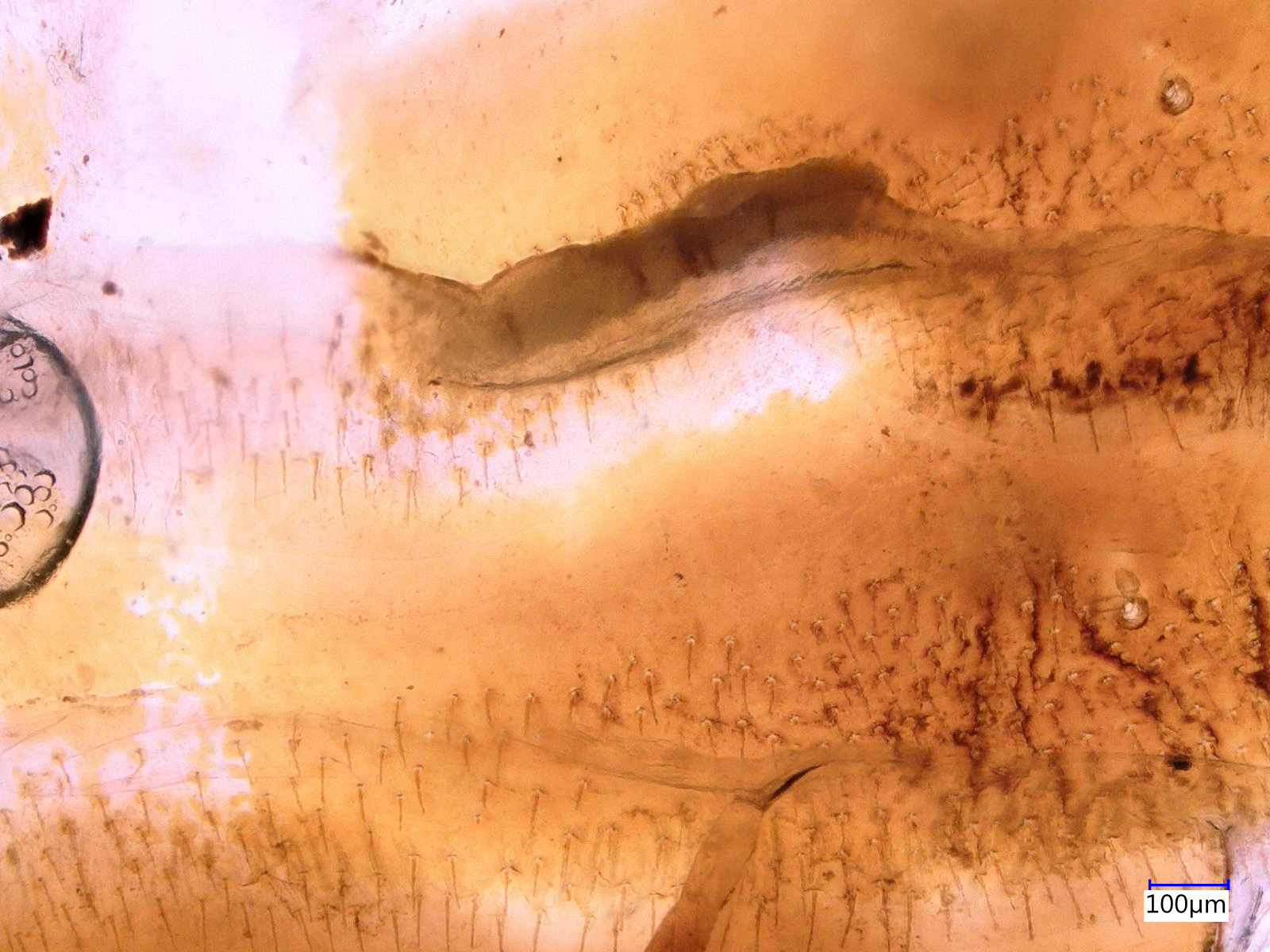


**Figure S1** – Mating scars (red arrows) on female ectospermalege.

**Figure S2** – Melanization of mating scars over time represented the proportion of mating scars observed 30 minutes, 1 hour, 12 hours, 24 hours, 48 hours and five days after a single mating.
